# Sri Lanka in global medical research: a scientific analysis of the Sri Lankan research output during 2000-2009

**DOI:** 10.1186/1756-0500-5-121

**Published:** 2012-02-24

**Authors:** Priyanga Ranasinghe, Ranil Jayawardena, Prasad Katulanda

**Affiliations:** 1 Diabetes Research Unit, Department of Clinical Medicine, Faculty of Medicine, University of Colombo, Colombo, Sri Lanka; 2 School of Human Movement Studies, Institute of Health and Biomedical Innovation, Queensland University of Technology, Brisbane, QLD, Australia

**Keywords:** Sri Lanka, Medical research, Publication, Analysis

## Abstract

**Background:**

Scientific research is an essential component in guiding improvements in health systems. There are no studies examining the Sri Lankan medical research output at international level. The present study evaluated the Sri Lankan research performance in medicine as reflected by the research publications output between years 2000-2009.

**Methods:**

This study was based on Sri Lankan medical research publication data, retrieved from the SciVerse Scopus^® ^from January 2000 to December 2009. The process of article selection was as follows: Affiliation - 'Sri Lanka' or 'Ceylon', Publication year - 'January 2000 to December 2009' and Subject area - 'Life and Health Sciences'. The articles identified were classified according to disease, medical speciality, institutions, major international collaborators, authors and journals.

**Results:**

Sri Lanka's cumulative medical publications output between years 2000-2009 was 1,740 articles published in 160 different journals. The average annual publication growth rate was 9.1%. Majority of the articles were published in 'International' (n = 950, 54.6%) journals. Most articles were descriptive studies (n = 611, 35.1%), letters (n-345, 19.8%) and case reports (n = 311, 17.9%). The articles were authored by 148 different Sri Lankan authors from 146 different institutions. The three most prolific local institutions were Universities of; Colombo (n = 547), Kelaniya (n = 246) and Peradeniya (n = 222). Eighty four countries were found to have published collaborative papers with Sri Lankan authors during the last decade. UK was the largest collaborating partner (n = 263, 15.1%).

Malaria (n = 75), Diabetes Mellitus (n = 55), Dengue (n = 53), Accidental injuries (n = 42) and Lymphatic filariasis (n = 40) were the major diseases studied. The 1,740 publications were cited 9,708 times, with an average citation of 5.6 per paper. The most cited paper had 203 citations, while there were 597 publications with no citations. The Sri Lankan authors' contribution to the global medical research output during the last decade was only 0.086%.

**Conclusion:**

The Sri Lankan medical research output during the last decade is only a small fraction of the global research output. There it is a necessity to setup an enabling environment for research, with a proper vision, support, funds and training. In addition, collaborations across the region need to be strengthened to face common regional health challenges.

## Background

Sri Lanka, is a rapidly developing island nation in the Indian subcontinent that has a population of nearly 20.5 million [1]. The country is presently facing a double burden of both communicable and non-communicable diseases [2]. Communicable diseases such as dengue, leptospirosis, tuberculosis, diarrhoeal diseases and acute respiratory infection are still important causes of morbidity and mortality [3]. Non-communicable diseases too have increased rapidly, especially cardiovascular diseases, cancers, diabetes, and tobacco, alcohol and substance abuse; while intentional pesticide poisoning has also been a problem for many years [4]. Mental health disorders are also common, with a particularly high suicide rate (around 6000 per year) [5]. In addition injuries, including road traffic accidents, are a major cause of hospital admission and mortality [2]. In Sri Lanka, health care services are mostly provided through a network of government operated public health care services at different levels. Most people live within 5 km of a health facility. At present there are 1,042 public hospitals in the country, with a bed strength of nearly 70,000, around 13,000 medical doctors and over 30,000 nurses work at these institutions [1,6]. In 2009, 4.0% of the GDP was spent on health care expenditure [2]. There are eight medical schools in Sri Lanka, dispersed in five provinces and operational under government universities, producing nearly 1,000 medical doctors annually.

Scientific research is an essential component in guiding improvements in health systems and development of new initiatives [7]. Sri Lanka is recognized internationally for its good health indicators at a quite low level of GDP (approx 2,500) and is at the forefront in the South Asian region in providing quality health services [8]. However, gaps remain in local evidence required to develop guidelines to address nationally important health problems such as dengue viral infection, diabetes, ischaemic heart disease and injuries due to road traffic accidents. Furthermore, even when technical knowledge is available, political commitment, managerial competencies, and incentives for changing behaviour within health systems are often lacking in Sri Lanka, similar to other countries of the South Asian region [9]. Hence, scientific research is important to identify solutions to the challenges faced by the Sri Lankan health care system, in order to refine and tailor local health care practices to suit national requirements. Therefore, frequent assessment of the national medical research output is necessary to identify deficiencies and plan improvements.

There are only few quantitative studies scientifically exploring the Sri Lankan medical or biomedical research output. Ranasinghe et al, analysed the process and costs of publishing medical journals in Sri Lanka, they identified 44 solely medical journals published in Sri Lanka, of which 28 were in print at the time of the study [10]. The Ceylon Medical Journal was the only journal indexed in PubMed, Web of Science, Scopus and other popular medical databases. The remaining journals were mainly in circulation within the local scientific community and most were open access [10]. The total number of articles published in the 28 active local medical journals in year 2009 when standardized to the national population was 22.2 per million [10]. This demonstrates a significant improvement from the national population standardized figure of 2.95 per million in year 2000 [11]. However, the actual population standardized national figure mentioned above for 2009 would be much higher as it does not include the publications in non-local/international journals.

At present there are no studies examining the Sri Lankan medical research output at international level. The main objective of this study is to analyze the Sri Lankan research performance in medicine in the national and global contexts, as reflected by its publication output during 2000-2009. The objectives of this study were: (i) to study the evolution of the Sri Lankan medical research output during the last decade, (ii) to examine the publications' productivity and impact of leading institutions in Sri Lanka, (iii) to identify the patterns of international collaboration and major collaborative partners, (iv) to report the distribution of Sri Lankan medical publications by disease and medical speciality, (v) to identify the most prolific authors, (vi) to examine the patterns of research communication in most productive journals and (vii) to compare the national research output with other countries of the region and world.

## Methods

This study was based on the Sri Lankan medical research publication data, retrieved from the SciVerse Scopus^® ^(Elsevier Properties S.A, USA) database for 10 years from 1st January 2000 to 31st December 2009. The process used in article selection was as follows: affiliation - 'Sri Lanka' or 'Ceylon', Publication year - '1st January 2000 to 31st December 2009' and Subject area - 'Life and Health Sciences'. Conference proceedings, trade publications, books and book series were excluded. The articles identified were classified according to research output based on; disease, medical speciality, institutions, major international collaborators, authors and journals.

Journals were classified either as 'International' or 'Regional' (Asian) based on journal publishers' details available at the Scopus^® ^database. Studies were classified based on study design into following categories by perusal of individual abstracts; 'Audits', 'Case-Control', 'Case reports', 'Cohort analysis', 'Descriptive', 'Experimental', 'Letters', 'Pre-clinical trials' and 'Randomized control trials'. In order to identify extent of collaborations with other countries, Sri Lankan authors of individual articles were grouped based on authorship of the article as either 'Corresponding author' or 'Co-author'. Individual articles were also grouped into diseases and medical specialities by perusal of abstracts. This entire process was conducted by two independent reviewers and the final group of articles to be included in the review was determined after an iterative consensus process amongst the reviewers.

The global research output was evaluated by total number of research papers from 'Life and Health Sciences' published in Scopus^® ^between years 2000-2009. The global research output on individual diseases were evaluated by using disease names in search field 'Article abstract, title or keywords' from 'Life and Health Sciences' published in Scopus^® ^between years 2000-2009. The research output of five leading countries each from different regions of the world were also identified by a similar method. Population standardized figures for Sri Lanka were obtained based on population data from the Department of Census and Statistics Sri Lanka [1]. Population standardized figures for other countries were based on latest population data available for the respective countries. Citations for the articles identified using the above search criteria were evaluated using the Scopus^® ^Citation Analyzer up to 31st December 2011. Self citations were excluded to evaluate true global impact. In order to evaluate factors determining citations of national authors the articles were grouped based on number of citations as follows; No citations, 1-10 citations, 11-50 citations, 51-100 citations and > 100 citations. The characteristics of most high-cited papers (> 100 citations) from Sri Lanka were evaluated. To evaluate the impact of Sri Lankan medical publication, the h-index of the publications was calculated by Scopus^® ^h-index calculator. The h-index is an index that attempts to measure both the productivity and impact of authors, research groups and journals [12]. We calculated the h-index of individual Sri Lankan authors and research institutions/groups with most publications.

## Results

**Figure 1:**
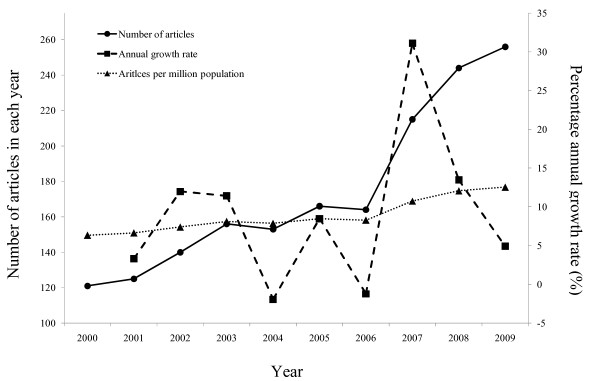
**Annual research publications output 2000-2009**.

Sri Lanka's cumulative medical publication output between years 2000-2009 consisted of 1,740 papers. The number of publications increased from 121 in year 2000 to 256 in year 2009, with an average annual publication growth rate of 9.1% and an h-index of 43. Figure 1 illustrates the secular trends for national research publications from year 2000-2009.

The articles were published in 160 different journals and only two 'Sri Lankan' journals were indexed in Scopus, the Ceylon Medical Journal and the Journal of the National Science Foundation of Sri Lanka, 20 journals were 'Regional' and the remaining 138 journals were 'International'. The two 'Sri Lankan' journals accounted for 35.7% of the publications, while a majority of the articles were published in 'International' (n = 950, 54.6%) journals and the remaining articles were published in 'Regional' journals (n = 169, 9.7%). However when considering the individual journals the Ceylon Medical Journal (n = 600, 34.5%) accounted for a majority of the publications, followed by the Transactions of the Royal Society of Tropical Medicine and Hygiene (n = 42, 2.4%), Lancet (n = 30, 1.7%), British Medical Journal (n = 21, 1.2%) and Southeast Asian Journal of Tropical Medicine and Public Health (n = 21, 1.2%). Publications in 'International' journals showed a higher growth rate during 2005-2009 (113.6%) than between 2000-2004 (84.8%). The publication growth rate of the two Sri Lankan journals during both time periods were negative and further declined from -14.5% in first half of decade (2000-2004) to -20.6 in the second half (2005-2009).

Majority of the articles published in the journals were descriptive studies (n = 611, 35.1%), letters (n-345, 19.8%) and case reports (n = 311, 17.9%). There were only 37 randomized controlled trials (2.1%) and 35 pre-clinical trials (animal studies) (2.0%), while 115 articles were systematic reviews (6.6%). The publication pattern of the Ceylon Medical Journal which carried majority of the publications was as follow; Case report (n = 194, 32.3%), descriptive studies (n = 193, 32.2%), letters (n = 178, 29.7%), systematic reviews (n = 17, 2.8%), randomized control trials (n = 3, 0.5%) and others (n = 15, 2.5%). Randomized controlled trials and pre-clinical animal studies had the highest citations rates (Table 1). However, the Sri Lankan author was the corresponding authors in only 56.8% Randomized Controlled Trials and 48.5% pre-clinical animal studies. In contrast in all the other study types the Sri Lankan author was the corresponding author in > 80% of the articles (Table 1). The articles have been authored by 148 different Sri Lankan authors. In the Ceylon Medical Journal and the Journal of the National Science Foundation of Sri Lanka the Sri Lankan author was the corresponding author in all articles published. The Sri Lankan author was the corresponding author in 63.3% (n = 708) and a co-author in 36.7% (n = 411) of the remaining 1,119 articles published in 'International' and 'Regional' journals. The most published authors were; de Silva, H.J. (n = 65), Eddleston, M. (n = 61), Buckley, N.A. (n = 49), Ratnasooriya, W.D. (n = 41) and Dawson, A.H. (n = 36) (see Additional file 1).

**Table 1:** Types of study and their relative impact

Type of study	Number (%)	Citation rate	h-index	Correspondence from Sri Lanka (%)
Descriptive studies	611(35.1%)	2.4	10	528(86.1%)

Letters	345(19.8%)	0.3	8	305(88.4%)

Case reports	311(17.9%)	0.6	9	296(95.2%)

Systematic reviews	115(6.6%)	7.7	26	107(93.0%)

Randomized Controlled Trials	37(2.1%)	18.1	26	21(56.8%)

Pre-clinical animal studies	35(2.0%)	9.5	13	17(48.5%)

The authors of the articles were from 146 different local and international institutions. The institutions engaged in research activities in medicine were classified into four categories; local medical faculties, local hospitals, other local research institutions and international institutions. The three most prolific local medical faculties in terms of research were from University of Colombo (n = 547), University of Kelaniya (n = 246) and University of Peradeniya (n = 222). However, there were no studies from recently established medical faculties at University of Rajarata (established in 2005) and Eastern University of Sri Lanka (established in 2006). The cumulative research output from 15 local hospitals were 286, which accounted for a 16.4% share of the total national publication output. The National Hospital of Sri Lanka, Colombo (n = 130), The Lady Ridgeway Hospital for Children, Colombo (n = 28) and Anuradhapura Teaching Hospital, Anuradhapura (n = 16) were the most productive local hospitals in terms of research publications. The three other local institutions with the highest research output were Medical Research Institute, Colombo (n = 37), Institute of Fundamental Studies, Kandy (n = 26) and The International Water Management Institute, Colombo (n = 26). The collaborating international institutes with the highest number of publications during the last decade were The Nuffield Department of Clinical Medicine, University of Oxford, Oxford, UK (n = 64), King's College, London, UK (n = 26) and Liverpool School of Tropical Medicine, Liverpool, UK (n = 20). University of Colombo had the highest h-index (h = 33), followed by University of Peradeniya, Kandy (h = 20), The National Hospital of Sri Lanka (h = 17) and University of Kelaniya, Ragama (h = 17).

Among the major international collaborative partners of Sri Lanka, as reflected in its international co-authored papers, 84 countries were found to have published collaborative papers with Sri Lankan authors during the last decade. United Kingdom was the largest collaborating partner between year 2000-2009 by contributing 15.1% (n = 263) of the publications share in Sri Lanka's total international collaborative papers in medicine, followed by the Australia (7.4%, n = 130), United States (5.1%, n = 89), India (3.9%, n = 69) and Japan (3.2%, n = 56). The citation rate of articles without any international collaboration was 0.8, where as in articles with international collaborations the citation rate was 5.3.

The Sri Lankan research output during the last decade was categorized in to different diseases; Malaria (n = 75), Diabetes Mellitus (n = 55), Dengue (n = 53), Accidental injuries (n = 42) and Lymphatic filariasis (n = 40) were the major diseases studied (Table 2). In terms of the global publications share, the contribution from Sri Lankan authors under the above diseases were as follows; Malaria (0.3%), Diabetes Mellitus (0.04%), Dengue (1.1%), Accidental Injuries (0.01%) and Lymphatic Filariasis (6.3%). The major medical specialities investigated were Microbiology (n = 201), Gynaecology and Obstetrics (n = 189), Parasitology (n = 150), Psychology (n = 150) and Surgery (n = 139). The greatest increase in research publications from first to second half of the decade was seen for HIV/AIDS, Tuberculosis, Chronic Kidney Disease (CKD), Dengue infection and Diabetes Mellitus. Similarly the medical specialities that has seen the highest increase in research productivity were; Pharmacology, Genetics, Nutrition, Endocrinology, Toxicology and Surgery (Table 2).

**Table 2:** Diseases and medical specialities with more than ten publications between years 2000-2009

Disease	Number of Publications			Sub-speciality	Number of Publications		
			
	2000-2004	2005-2009	% increase*		2000-2004	2005-2009	% increase*
Malaria	27	48	77.8	Microbiology	92	109	18.5

DM	16	39	143.8	Gyn & Obs	69	120	73.9

Dengue	11	42	281.8	Parasitology	58	92	58.6

Injuries	7	35	400	Psychiatry	62	88	41.9

Filariasis	17	23	35.3	Surgery	47	92	95.7

Snake bite	12	11	-8.3	Toxicology	37	88	137.8

HIV/AIDS	2	10	400	Phytomedicine	38	45	18.4

Tuberculosis	2	10	400	Endocrinology	18	51	183.3

CKD	1	10	900	Nutrition	9	35	288.9

Epilepsy	6	4	-33.3	FM	9	27	200

IHD	4	6	50	Neurology	13	23	76.9

				Medical Education	16	14	-12.5

				Genetics	5	21	320

				Pathology	10	16	60

				Pharmacology	3	19	533.3333

				Oncology	11	8	-27.2727

				Dermatology	6	10	66.7

*Percentage increase from first half to second half of decade; *DM *Diabetes Mellitus; *CKD *Chronic Kidney Disease; *IHD *Ischaemic Heart Diseases; *FM *Forensic Medicine

The 1,740 publications were cited 9,708 (excluding 1,389 self citations) times, with an average citation of 5.6 per paper. A majority of the citations were from US (2,176), UK (1,326) and India (1,006), while Sri Lankan authors have cited the publications 733 times. The most cited paper had 203 citations, while there were 597 (34.3%) publications with no citations. The documents were grouped based on citations; 1-10 citations (n = 920, 52.9%), 11-50 citations (n = 193, 11.1%), 51-100 citations (n = 23, 1.3%) and > 100 citations (n = 7, 0.4%). The characteristics of the ten most high-cited papers (> 100 citations) from Sri Lanka were also evaluated, 4 of them were review articles, 2 were descriptive studies and 1 was a genetic study. The 7 high-cited papers, all involved international collaboration (2 bilateral and 5 multilateral). The Sri Lankan author was the corresponding author for 3 papers. Four of the papers were on toxicology and poisoning and two papers were on Dengue viral infections. The authors of these high-cited papers were affiliated to 6 Sri Lankan institutions, including 5 papers from University of Colombo and 1 paper each from University of Sri Jayewardenapura, University of Jaffna, University of Kelaniya, Medical Research Institute, Colombo and Kandy Teaching Hospital, Kandy. These papers have appeared in 6 different journals, including 2 papers in Lancet, 1 paper each in Journal of the American Medical Association, Trend in Parasitology, Postgraduate Medical Journal and Human Molecular Genetics.

The number of research papers related to medicine published internationally during the same time period was 2,018,917. Sri Lankan authors contributed to only 0.086% of the global medical research output, a figure that has remained relatively static from 0.078% in 2000 to 0.089% in 2009. The average annual population standardized publication rate for Sri Lanka was 8.4. Table 3 lists the top five countries in terms of number of publications from different regions of the world. The Sri Lankan contribution to global medical publications output during the last decade is one of the lowest.

**Table 3:** The top five countries with highest publications from different regions of the world

Countries	Publications between 2000 and 2009		
	
	Number	% of global publications	Average per million population
Africa			
South Africa	9,319	0.46%	18.4
Egypt	5,058	0.25%	6.2
Nigeria	3,692	0.18%	2.3
Tunisia	1,341	0.07%	12.6
Kenya	1,041	0.05%	2.7

Asia			
Japan	111,929	5.5%	87.6
India	49,064	2.4%	4.0
Turkey	46,584	2.3%	63.2
China	32,464	1.6%	2.4
Taiwan	27,613	1.4%	119.5

Australia			
Australia	62,970	3.1%	279.9
New Zealand	11,265	0.6%	256.0

Europe			

United Kingdom	193,898	9.6%	311.2
Germany	89,761	4.4%	109.9
Italy	80,963	4.0%	133.4
France	52,606	2.6%	79.9
Netherlands	50,274	2.5%	301.0

North America			
USA	585,526	29.0%	187.4
Canada	76,722	3.8%	222.4
Mexico	5,438	0.3%	4.8
Jamaica	798	0.03%	29.6
Cuba	469	0.02%	4.2

South America			
Brazil	22,558	1.1%	11.8
Argentina	4,437	0.2%	11.1
Chile	2,006	0.1%	11.6
Venezuela	938	0.04%	3.2
Peru	689	0.03%	2.3

## Discussion

This is the first study evaluating the national medical research output from Sri Lanka. Our results demonstrate that the number of medical publications from Sri Lanka in indexed journals remain relatively low during the last decade with only a negligible contribution of 0.086% to the global medical research output. The national medical research output is also comparatively lower than other regional and international countries. Sri Lanka has the best health indicators in the South Asian region. However, the medical research output from Sri Lanka during the last decade is much lower than from other South Asian countries such as India (49,064), Pakistan (7,362) and Bangladesh (2,640). In 2006 Sri Lanka's Research & Development (R & D) expenditure was 0.2% of GDP (per capita GDP approx 2,500) and that of India 1.1%, Pakistan 0.3% and Nepal 0.2%, while East-Asian countries invested 1.5-2.5% of GDP on R & D [13]. In addition the Sri Lanka National Research Council data shows that only 317 (approx 15 per million population) Sri Lankan scientist were published in the Science Citation Index journals in 2006 [13]. Scientific research is an essential cornerstone in the evolution of health care systems and development of new initiatives [7]. While research is important to tackle locally prevalent health issues, dissemination of knowledge via internationally accessible publications such as indexed journals to regional and international countries helps to share knowledge and identify novel solutions to regionally important problems. Hence, it is important to critically evaluate the probable reasons that contribute towards the relatively low medical research output from Sri Lanka.

The total number of publications in medicine from Sri Lanka during the last decade was 1,740. A study evaluating local Sri Lankan medical journals has shown that the number of articles published in non-indexed Sri Lankan journals in 2009 is 406 [10], while in the same year our results demonstrate that Sri Lankan authors have published only 256 papers in indexed journals. Hence, it is evident that over 60% of the national research output in 2009 has been published in non-indexed local journals, limiting their availability to an international audience. Publications in 'International' journals have increase dramatically by over 250% during the last decade. However, in contrast the publication growth rate of indexed Sri Lankan journals during same period has been -21.7% and further declining from first to second half of the decade. The high production costs possibly contributes towards the observed decline in the number of publications in the indexed Sri Lankan journals [10]. Hence, with higher competition for publication in the two indexed local journals the local researches have been forced to publish their research in non-indexed local journals. These journals have a relatively small readership and are strapped financially; making it difficult to keep up with changing technology [14]. A possible solution would be for established indexed international journals that are at the center of scientific publishing to help smaller journals in developing countries grow by sponsoring, redirecting articles and soliciting specific articles from them for publication which will also result in the international dissemination of knowledge [14].

The citation rate per article excluding self-citations for the published articles was 5.6. A study conducted using the Web of Science database in 2010 has shown that the citation rate for Sri Lanka between years 2005-2009 was 2.7 [15]. The citation rate of 2.6 (excluding self-citations) for the same time period using our data was comparable to these findings. This citation rate was comparable to that of India (2.7), however it was much lower than the citation rates of other more developed regional countries such as China (4.2), Japan (4.6) and Singapore (5.9). It is also very clearly seen that collaborative partnerships with other countries significantly increases the citations rates, and hence the global impact of the publication. Majority of the articles published by Sri Lankan authors were descriptive studies (35.1%), letters (19.8%) and case reports (17.9%), with only 2.1% being randomized controlled trials. The Ceylon Medical Journal which carried majority of the publications also showed a similar pattern of publication. This could be another reason for the lesser number of publications by Sri Lankan authors in international indexed journals. Studies on major biomedical journals have shown that submitted manuscripts are more likely to be published if they have an RCT study design, if the corresponding author lives in the same country as that of the publishing journal and carries a larger sample size [16].

The Sri Lankan medical research output during the last decade has been mainly focused on nationally important diseases such as; Malaria, Diabetes Mellitus, Dengue and Accidental injuries. The publications trends have also reflected on changes in national disease patterns as evident by the high growth rates of publications on accidental injuries, Dengue, Diabetes Mellitus and Chronic Kidney Disease, which are important national health concerns at present [2]. It is interesting to note that Sri Lanka shares a significant percentage of the global research output on diseases such as Dengue (1.1%) and Lymphatic Filariasis (6.3%). The average number of citations per article for article on Dengue (12.1) and Lymphatic Filariasis (9.2) was significantly higher than that of other articles (6.3). Dengue and Lymphatic Filariasis are both included in the Neglected Tropical Diseases list by the World Health Organization. The neglected diseases are a group of tropical infections which are especially endemic in low-income populations in developing regions of Africa, Asia, and the Americas. Hence it is evident that Sri Lanka plays a leading role in research in fields that are important for developing low socio-economic countries. However, the research activities on other nationally important health issues such as ischaemic heart disease, hypertension, obesity and infections such as leptospirosis are very minimal. This discrepancy could be due to lack of funding and proper allocation of health resources, which is a common problem faced by many developing countries such as Sri Lanka. The Global Forum for Health Research emphasised the need to strengthen research capacity in developing countries to redress the "10/90" gap--that only 10% of all global health research funding was allocated to 90% of the world's burden of preventable mortality [17]. In many South Asian countries, such as Sri Lanka inadequate funding is the main barrier to health research. Research in South Asia is often viewed as expenditure than an investment and this has resulted in the need to search for research funding from external donors. This is evident from the pattern of international collaboration seen in Sri Lankan publications. A majority of collaborations have been with developed countries with readily available research funding such as the United Kingdom, Australia, United States, Japan, Switzerland, Denmark and Germany, while only 6.1% of collaborations have been with other regional South Asian countries such as India, Pakistan, Bangladesh and Nepal. Hence there is also a need to strengthen the collaborations across the region in order to face common challenges efficiently.

The global impact of Sri Lankan medical research also seems to be relatively low. A majority of the publication had less than ten citations of which over 30% had zero citations. It has been shown that citation frequency depends on journal impact factor, country of study and topic of the study [18]. Majority of the Sri Lankan publications were published in the Ceylon Medical Journal (34.4%), with an h-index of 10, which is significantly lower than the h-index high-impact international journals such as NEJM (h = 571), Lancet (h = 421) and BMJ (h = 243). In contrast a majority of the high-cited papers were multi-national collaborations and published in high-impact and high-circulation journals. Hence, while it is important to increase the number of research publications from Sri Lanka, it is equally important to identify and find solutions to the factors that contributes towards this lower level of global impact of Sri Lankan research.

The study was conducted in Scopus^®^, which has been shown to have a slight edge over other indexes such as Web of Science and Google Scholar for articles related to 'life sciences' [19]. Scopus^® ^offers an interactive environment for easy-tracking, storage and classification of research publications. A search performed in Web of Science using the same search criteria resulted in 1,467 articles. This discrepancy could be because Scopus^® ^includes a more expanded spectrum of journals than PubMed and Web of Science [20]. A potential limitation in Scopus^® ^is that it includes cited references only from 1995 onwards. This limitation is overcome in the present study as it analyses only articles from years 2000 to 2009. The use of Web of Science has been the standard for citation analysis, yet studies have shown that Scopus^® ^does offer a 20% more citation analyses in any given period than Web of Science [20]. However, these additional citations are derived from vague journals in non-English languages, a limitation even present to an extent in the Web of Science [20]. In addition studies have shown that the citation analysis in Scopus^® ^is much faster and more reproducible than the citation analysis in Web of Science [20]. Hence, although with certain limitations the Scopus^® ^database suited the requirements of the present study

In addition, we used the h-index for measuring relative impact of published work. Both quantity and impact of publications are taken into account when calculating the h-index, but the number of publications plays a very important role, since it is the maximum h-index an author can obtain [21]. The h-index tends to underestimate the achievement of scientists with a "selective publication strategy", that is, those who do not publish a high number of documents but who achieve a very important international impact that is; the best correlation is found with absolute indicators of quantity [21]. The use of h-index as a single indicator for the assessment of the impact of published work may not be adequate. Hence, we have complemented the h-index with citation rates to gauge an accurate estimation of the impact of published work.

## Conclusions

The Sri Lankan medical research output during the last decade is relatively small, with minimal contribution to the global research output. Hence there it is an urgent necessity to setup an enabling environment for research, with a proper vision, institutional support, adequate funds and appropriate training. In addition, collaboration across the region needs to be strengthened to efficiently face future regional health care challenges.

## Competing interests

The authors declare that they have no competing interests.

## Authors' contributions

All authors were involved in study design, data collection and analysis. PR drafted the initial manuscript. The manuscript was critically reviewed by MARJ and PK. All authors read and approved the final manuscript.

## Supplementary Material

Additional file 1 **The ten most published authors, journals and institutions in Sri Lanka**. Click here for file
