# Supplementary material for: Sri Lanka in global medical research: a scientific analysis of the Sri Lankan research output during 2000-2009
Source: BMC Res Notes. 2012 Feb 24;5:121. doi: 10.1186/1756-0500-5-121 (PMC3305378; doi:10.1186/1756-0500-5-121)
Supplement: Additional file 1 — The ten most published authors, journals and institutions in Sri Lanka. [file 1756-0500-5-121-S1.DOC]

**Table 3:** The ten most published authors, journals and institutions in Sri Lanka

|  | Journals | Authors | Institutions |
| --- | --- | --- | --- |
| 1 | Ceylon Medical Journal (n=600, h=10) | de Silva, H.J. (n=65, h=12) | University of Colombo, Colombo, LK (n=547, h=33) |
| 2 | Transactions of Royal Society of Tropical Medicine & Hygiene (n=42, h=10) | Eddleston, M. (n=61, h=21) | University of Kelaniya, Ragama, LK (n=246, h=17) |
| 3 | Lancet (n=30, h=14) | Buckley, N.A. (n=49, h=16) | University of Peradeniya, Kandy, LK (n=222, h=20) |
| 4 | British Medical Journal (n=21, h=7) | Ratnasooriya, W.D. (n=41, h=10) | The National Hospital of Sri Lanka, Colombo, LK (n=130, h=17) |
| 5 | Southeast Asian Journal of Tropical Medicine and Public Health (n=21, h=6) | Dawson, A.H. (n=36, h=11) | University of Sri Jayewardenepura, Nugegoda, LK (n=125, h=14) |
| 6 | Journal of the National Science Foundation of Sri Lanka (n=20, h=2) | Karunaweera, N.D. (n=35, h=9) | University of Ruhuna, Galle, LK (n=78, h=12) |
| 7 | Annals of Tropical Medicine and Parasitology (n=19, h=7) | Sheriff, M.H.R. (n=34, h=14) | Medical Research Institute, Colombo, LK (n=37, h=9) |
| 8 | Clinical Toxicology (n=19, h=5) | Kuruppuarachchi, K.A. (n=31, h=3) | Lady Ridgeway Hospital for Children, Colombo, LK (n=28, h=7) |
| 9 | British Journal of Psychiatry (n=17, h=6) | Wickremasinghe, A.R. (n=30, h=9) | Institute of Fundamental Studies, Kandy, LK (n=26, h=7) |
| 10 | Pharmaceutical Biology (n=16, h=5) | Lamabadusuriya, S.P. (n=30, h=5) | International Water Management Institute, Colombo, LK (n=26, h=12) |
